# Generalized neurocognitive impairment in individuals at ultra‐high risk for psychosis: The possible key role of slowed processing speed

**DOI:** 10.1002/brb3.1962

**Published:** 2021-01-23

**Authors:** Lasse Randers, Jens Richardt Møllegaard Jepsen, Birgitte Fagerlund, Dorte Nordholm, Kristine Krakauer, Carsten Hjorthøj, Birte Glenthøj, Merete Nordentoft

**Affiliations:** ^1^ Copenhagen Research Center for Mental Health ‐ CORE Mental Health Center Copenhagen Copenhagen University Hospital Copenhagen Denmark; ^2^ Center for Clinical Intervention and Neuropsychiatric Schizophrenia Research (CINS) Mental Health Center Glostrup Copenhagen University Hospital Glostrup Denmark; ^3^ Faculty of Health and Medical Sciences Department of Clinical Medicine University of Copenhagen Copenhagen Denmark; ^4^ Center for Neuropsychiatric Schizophrenia Research (CNSR) Mental Health Center Glostrup Copenhagen University Hospital Glostrup Denmark; ^5^ Child and Adolescent Mental Health Center Mental Health Services Capital Region of Denmark Copenhagen University Hospital Denmark; ^6^ Faculty of Social Sciences Department of Psychology University of Copenhagen Copenhagen Denmark; ^7^ Functional Imaging Unit Department of Clinical Physiology, Nuclear Medicine and PET Copenhagen University Hospital Rigshospitalet Glostrup Denmark; ^8^ Faculty of Health and Medical Sciences Department of Public Health Section of Epidemiology University of Copenhagen Copenhagen Denmark

**Keywords:** at‐risk mental state, clinical high risk, cognition, neuropsychology, schizophrenia

## Abstract

**Objective:**

Widespread neurocognitive impairment is well‐established in individuals at ultra‐high risk (UHR) for developing psychoses, but it is unknown whether slowed processing speed may underlie impairment in other neurocognitive domains, as found in schizophrenia. The study delineated domain functioning in a UHR sample and examined if neurocognitive slowing might account for deficits across domains.

**Methods:**

The cross‐sectional study included 50 UHR individuals with no (*n* = 38) or minimal antipsychotic exposure (*n* = 12; mean lifetime dose of haloperidol equivalent = 17.56 mg; *SD* = 13.04) and 50 matched healthy controls. Primary analyses compared group performance across neurocognitive domains before and after covarying for processing speed. To examine the specificity of processing speed effects, post hoc analyses examined the impact of the other neurocognitive domains and intelligence as covariates.

**Results:**

UHR individuals exhibited significant impairment across all neurocognitive domains (all *p*s ≤ .010), with medium to large effect sizes (Cohen's *d*s = −0.53 to −1.12). Only processing speed used as covariate eliminated significant between‐group differences in all other domains, reducing unadjusted Cohen's *d* values with 68% on average, whereas the other domains used as covariates averagely reduced unadjusted Cohen's *d* values with 20% to 48%. When covarying each of the other domains after their shared variance with speed of processing was removed, all significant between‐group domain differences remained (all *p*s ≤ .024).

**Conclusion:**

Slowed processing speed may underlie generalized neurocognitive impairment in UHR individuals and represent a potential intervention target.

## Graphical abstract

The study delineated neurocognitive domain functioning in 50 ultra‐high risk individuals and 50 healthy controls, examining if slowed processing speed might contribute significantly to impairment in other neurocognitive domains in the ultra‐high risk group. The results showed that the ultra‐high risk individuals exhibited significant impairment across all neurocognitive domains, and processing speed was able to account for the significant between‐group differences in all other domains.

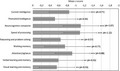

## 1 INTRODUCTION

Individuals at ultra‐high risk (UHR) for schizophrenia and other psychoses exhibit neurocognitive impairment across more or less all domains, with small to medium effect sizes typically reported in meta‐analyses (e.g., Bora et al., 2014; Giuliano et al., 2012). Slowed speed of processing has yielded the highest effect size (Hedges' *g* = −0.43) across neurocognitive domains in the largest meta‐analysis (Hauser et al., 2017) to date, and several UHR studies have suggested prominence of this domain (e.g., Carrión et al., 2011; Keefe et al., 2006; Kelleher et al., 2013; Metzler et al., 2014), just as impairment on processing speed may be predictive of transition to psychosis (Riecher‐Rössler et al., 2009) and specifically the development of schizophrenia (Velthorst et al., 2019). However, despite this interest in neurocognitive slowing, its potential key role in UHR individuals’ broader neurocognitive impairment remains understudied and unclear.

Processing speed refers to how fast neurocognitive operations are executed and represents a wide‐reaching neurocognitive domain that may underlie functioning in many other domains (Dickinson & Gold, 2008b; Salthouse, 1996). As a general processing constraint, it may impose limits on an array of processing operations, for instance by reducing the number of times an item is rehearsed during memory encoding (Hartman et al., 2003). Processing speed may be described as a multidimensional domain that includes several basic, relatively simple neurocognitive components, including perceptual and motor functions, and it invariably emphasizes the velocity of information processing (Nuechterlein et al., 2004). In terms of psychometrics, this domain is generally measured by quantifying the number of correct responses made while performing a task in a finite amount of time. Among the most common types of instruments used to measure processing speed are digit symbol coding tasks (Dickinson et al., 2007), but there is no agreed consensus as to what the specific components of this domain may be (Low et al., 2017). A specific component often considered is motor speed, as it may be measured with the Token Motor Task of the Brief Assessment of Cognition in Schizophrenia (BACS) that requires the coordinated use of both hands (Keefe et al., 2004). As another example, finger tapping tests have been applied to measure the tapping speed of the index fingers (Reitan & Wolfson, 1993). Another specific component of processing speed often considered is cognitive speed that has been measured using nonmotor inspection time tasks. Such tasks measure the shortest target exposure duration needed to make a reliable perceptual discrimination without having to produce a psychomotor response (Low et al., 2017) and may thus index speed of apprehension (Kranzler & Jensen, 1989). Yet another component of processing speed having been considered is response selection that refers to the process of mapping stimuli to specific motor responses and decision making (Woodward et al., 2013). In addition, factor analytical studies indicate that verbal fluency tasks which focus on rapid spontaneous generation and articulation of words under restricted conditions typically load on processing speed (Nuechterlein et al., 2004). Verbal fluency tasks include, for example, controlled oral word association tests (Keefe et al., 2004). In schizophrenia, a substantial generalized neurocognitive impairment is well‐established, with slowed speed of processing likely being a core deficit (Dickinson et al., 2007). Processing speed has yielded the highest meta‐analytic effect size (*g* = −1.25) across all neurocognitive domains in this disorder (Schaefer et al., 2013), and several studies indicate that impairment in an array of neurocognitive domains may reflect reduced speed of processing to a significant degree in individuals with schizophrenia (e.g., Andersen et al., 2013; Rodríguez‐Sánchez et al., 2007). To our knowledge, the potential influence of slowed processing speed on deficits in a broad range of other neurocognitive domains has not previously been examined in the UHR population (Frommann et al., 2011; Koutsouleris et al., 2010). Thus, it remains to be investigated whether two cardinal features of neurocognition in schizophrenia, that is, a generalized deficit profile with slowed neurocognitive speed at its core, also characterize neurocognitive functioning of UHR individuals. This examination may provide insight into underlying mechanisms of broader neurocognitive impairment in UHR individuals and therefore be important for designing appropriate neurocognitive assessment and treatment (Dickinson & Harvey, 2009).

### 1.1 Aims of the study

This cross‐sectional study was designed to delineate neurocognitive domain functioning in UHR individuals compared to demographically matched healthy controls. The current aim was to clarify the potential key role of slowed processing speed for other neurocognitive domains. First, we examined the hypothesis that UHR individuals were characterized by a generalized deficit profile, with small to medium effect sizes across domains. Second, we explored the hypothesis that decreased processing speed accounted for significant between‐group differences in other domains.

## 2 METHODS

### 2.1 Participants and recruitment

The sample consisted of 50 UHR individuals and 50 healthy controls who all participated in the Prodromal Project, a Danish case–control research project on individuals at UHR of first‐episode psychosis. Inclusion period was September 2009 to August 2014. UHR individuals were referred to the Research Unit, Mental Health Center Copenhagen, from psychiatric in‐ and outpatient facilities in the Copenhagen catchment area. Healthy controls living in the same geographical area as the UHR individuals were recruited via a website for study participants and received payment for participation. They were matched one‐to‐one with UHR individuals on sex, age (within two years), parental socioeconomic status (total household income and highest parental education combined), and race/ethnicity (White/Asian/Mixed White‐Asian). Inclusion and exclusion criteria are listed in Table 1. Clinical, functional, and cognitive data on part of the sample have previously been reported (Dannevang et al., 2018; Krakauer et al., 2017, 2018; Madsen et al., 2018; Nordholm et al., 2016, 2018; Randers, Fagerlund, et al., 2020; Randers, Jepsen, et al., 2020). The Prodromal Project was approved by the Regional Ethics Committee of the Capital Region of Denmark (H‐D‐2009‐013) as well the Danish Data Protection Agency (2014‐41‐2861). It was carried out in accordance with the Declaration of Helsinki II, and participants signed informed consent.

**TABLE 1:** Inclusion and exclusion criteria

A. General inclusion criteria for the ultra‐high risk individuals
1. Being able to give informed consent 2. Being between 18–40 years old 3. Being help‐seeking 4. Being able to understand and speak Danish fluently
B. Membership of at least one of the following ultra‐high risk groups
1. Vulnerability (Trait and State Risk) Group: Individuals meeting diagnostic criteria for schizotypal personality disorder according to the DSM‐IV‐TR, and/or individuals having a first‐degree relative with a history of psychotic disorder
2. Attenuated Psychotic Symptoms (APS) Group: Individuals having experienced sub‐threshold (intensity or frequency) positive psychotic symptoms within the past year
3. Brief Limited Intermittent Psychotic Symptoms (BLIPS) Group: Individuals having experienced frank psychotic symptoms during the past year, but symptoms having resolved spontaneously (without antipsychotic medication) within one week
In all three groups, a functional impairment criterion must be met, as assessed with the SOFAS; symptoms must be associated with a significant drop in functioning within the past year (a SOFAS score at a minimum of 30% below previous level of functioning and sustained for at least one month) or a sustained low level of functioning during at least the last year (a SOFAS score of 50 or less continuously)
C. Exclusion criteria for the ultra‐high risk individuals
1. Past history of a treated or untreated psychotic episode of one week's duration or longer 2. Organic brain disease, for example, epilepsy, inflammatory brain disease 3. Abnormal thyroid function test results >10% above or below the limits of the normal range 4. Any physical illness with psychotropic effect, if not stabilized 5. Current treatment with any mood stabilizer or methylphenidate, or recreational use of ketamine 6. Past neuroleptic exposure equivalent to a total lifetime haloperidol dose of >50 mg. 7. Diagnosis of a serious developmental disorder, for example, Asperger's syndrome 8. Intelligence quotient <70 and a documented history of developmental delay or intellectual disability 9. Current aggression or dangerous behavior 10. Current suicidality or self‐harm 11. Current pregnancy 12. Current attenuated positive symptoms entirely explained by acute intoxication
D. General inclusion criteria for the healthy control individuals
1. Being able to give informed consent 2. Being between 18–40 years 3. Being able to understand and speak Danish fluently
E. Exclusion criteria for the healthy control individuals
1. Having a history of psychiatric disorder 2. Meeting criteria for any of the three ultra‐high risk groups listed in Section B 3. Meeting exclusion criteria for ultra‐high risk individuals listed in Section C 4. Having a first‐degree relative with a history of psychiatric disorder

Abbreviations: DSM‐IV‐TR, Diagnostic and Statistical Manual of Mental Disorders, Fourth Edition, Text Revision; SOFAS, Social and Occupational Functioning Assessment Scale.

### 2.2 Clinical and functional measures

To assess if participants met UHR criteria, the Comprehensive Assessment of At‐Risk Mental States (CAARMS) was applied (Yung et al., 2005) Psychiatric disorders were evaluated employing the Structured Clinical Interview for DSM‐IV‐TR Axis I Disorders (SCID‐I) (First et al., 2002) and Structured Clinical Interview for DSM‐IV Axis II Personality Disorders (SCID‐II) (First et al., 1997). Additional psychopathological and functional measures are listed in Table 3.

### 2.3 Neurocognitive measures

The Danish version of the National Adult Reading Test (Nelson & O'Connell, 1978), the Danish Adult Reading Test (DART), estimated premorbid intelligence. Current intelligence was estimated using four subtests (Vocabulary, Similarities, Block Design, and Matrix Reasoning) from the Wechsler Adult Intelligence Scale—Third Edition (WAIS‐III) (Wechsler, 1997). The omnibus battery also included the Danish version of the BACS (Keefe et al., 2004), selected tests from the Cambridge Neuropsychological Test Automated Battery (CANTAB) (Sahakian & Owen, 1992), and the Trail Making Test Parts A and B (TMT‐A and TMT‐B) (Reitan & Wolfson, 1993). In accordance with multiple UHR meta‐analyses (e.g., Hauser et al., 2017; Zheng et al., 2018) and individual studies (e.g., Eisenacher et al., 2018), the recommendations of the Measurement and Treatment Research to Improve Cognition in Schizophrenia (MATRICS) (Nuechterlein et al., 2004, 2008) were applied to categorize associated outcome variables into neurocognitive domains: speed of processing (including verbal fluency); attention/vigilance; reasoning and problem solving; verbal learning and memory; visual learning and memory; and working memory. Domains demonstrated satisfactory internal consistency (Cronbach's *α*s ≥ 0.73), as did a neurocognitive composite based on all six domains (*α* = 0.82). Table 2 outlines putative neurocognitive domains, tests, and associated outcome variables in detail.

**TABLE 2:** Neurocognitive test battery

Neurocognitive domain	Test and subtest	Outcome variable
Premorbid intelligence (estimated)	DART	Total number of words correctly pronounced^a^
Current intelligence (estimated)	WAIS‐III, four subtests: 1. Vocabulary 2. Similarities 3. Block Design 4. Matrix Reasoning	Total score for defining words^a^ Total score for identifying similarities between word pairs^a^ Total score for recreating pictured designs using blocks^a^ Total score for identifying patterns in pictured designs^a^
Speed of processing (Cronbach's *α* = 0.73, 5 outcome variables)	BACS Verbal Fluency: 1. Category Instances (Semantic Fluency) 2. Controlled Oral Word Association Test (Letter Fluency) BACS Token Motor Task BACS Symbol Coding Trail Making Test A	Total number of correct supermarket items generated Total number of correct F‐ and S‐words generated Total number of tokens placed correctly in container Total number of correct symbol‐digit pairs Total number of seconds to correctly connect all numbers in ascending order^d^
Working memory (Cronbach's *α* = 0.75, 4 outcome variables)	BACS Digit Sequencing Task CANTAB Spatial Working Memory (SWM) Trail Making Test B CANTAB Spatial Span	Total number of sequences of numbers correctly recalled with items in ascending order Total number of errors, including touching a box previously found empty in the same trial and touching a box with a token already found in a previous trial^b^ Total number of seconds to draw lines to correctly connect numbers and letters in alternating ascending order^b^ Longest sequential order of boxes changing color successfully recalled^a^
Verbal learning and memory (Single outcome variable)	BACS List learning	Total number of correct words recalled in five trials
Visual learning and memory (Single outcome variable)	CANTAB Delayed Matching to Sample (DMS)	Total number of correct visual patterns selected in all trials with delay^b^
Reasoning and problem solving (Cronbach's *α* = 0.74, 6 outcome variables)	BACS Tower of London CANTAB Stockings of Cambridge (SOC) CANTAB Spatial Working Memory (SWM) CANTAB Intra/Extra‐Dimensional Set Shift (IED) Trail Making Test B ‐ Trail Making Test A	Total number of problems solved in minimum moves Total number of problems solved in minimum moves^a^ Total number of times a new search is begun with the same box (strategy score)^b^ Total number of errors, that is, discriminating incorrectly between pairs of visual pattern stimuli (adjusted for number of completed stages)^d^ Total number of errors made in the extra‐dimensional stage^d^ Difference in seconds between time to complete Trail Making B and A^a^
Attention/vigilance (Cronbach's *α* = 0.73, 3 outcome variables)	CANTAB Rapid Visual Information Processing (RVP) CANTAB Reaction Time (RTI): 1. Simple Reaction Time 2. 5‐Choice Reaction Time	A', the signal detection measure of sensitivity to the target (combining number of hits and number of false alarms)^c^ Latency (response speed in milliseconds) with which press pad is released in response to onset of stimulus in a single location^d^ Latency (response speed in milliseconds) with which press pad is released in response to onset of stimulus in one of five locations^d^

Abbreviations: BACS, Brief Assessment of Cognition in Schizophrenia; CANTAB, Cambridge Neuropsychological Test Automated Battery; DART, Danish Adult Reading Test; WAIS‐III, Wechsler Adult Intelligence Scale‐Third Edition.

^a^ Square‐root transformation.

^b^ Lg10 transformation.

^c^ LnGamma transformation.

^d^ Reciprocal transformation.

### 2.4 Data analyses

Neurocognitive scores were converted to standard equivalents (*z*‐scores) based on means and standard deviations (*SD*s) of the healthy controls. Skewed and/or kurtotic distributions were approximated to normality by appropriate transformations (as specified in Table 2). In case of negatively skewed distributions, scores were first reflected, and reverse scoring was used when necessary to ensure that higher *z*‐score indicated better performance. If neurocognitive domains included more than one variable, contributing *z*‐scores were averaged by number of tests included, using equal weights. The domain z‐scores and the overall neurocognitive composite z‐score were standardized to obtain a mean of 0 and a *SD* of 1 in the healthy control group. Two UHR individuals did not complete the four WAIS‐III subtests, and extrapolation was therefore performed by replacing these missing data with the UHR group's mean estimated full scale intelligence *z*‐score. TMT‐A and TMT‐B were introduced after the study began; a total of 39 UHR individuals and all 50 healthy controls completed these tasks. For the UHR individuals missing the TMT outcome variables, substitution of group mean was likewise applied. No other neurocognitive data were missing. In the UHR group, outliers (BACS Tower of London [*n* = 1], BACS Verbal Learning and Memory [*n* = 1], CANTAB Rapid Visual Information Processing [RVP] [*n* = 2]) were truncated at −4 *SD*s to avoid undue distortion of group means and profile shape (Hochberger et al., 2016; Rhinewine et al., 2005). In the healthy control group, one outlier data point in the CANTAB RVP was removed and replaced with group mean.

For group comparison of demographic and clinical characteristics, Student's *t* test, Welch's *t* test, Mann–Whitney test, chi‐square test of independence, or Fisher's exact test was applied, as appropriate. Primary unadjusted analyses on neurocognitive functioning included one‐way multivariate analysis of variance (MANOVA) to examine the association between MATRICS domain performances and group relationship. A significant multivariate effect of group by domain was followed up by Student's or Welch's *t* test, as appropriate. A two‐way mixed ANOVA tested whether the shape of neurocognitive profile differed between groups. Group was the between‐subject factor, and domains were the within‐subject factor, with a two‐way significant effect of group by domain interaction indicating a deviation from flatness in the UHR individuals’ profile, that is, that some neurocognitive domain scores are significantly different from each other. To examine the influence of processing speed on between‐group neurocognitive domain variability, primary adjusted analyses included multivariate and univariate analyses of covariance (MANCOVAs and ANCOVAs), controlling group domain performances for speed of processing. To examine the specificity of processing speed effects, post hoc analyses examined the impact of the other neurocognitive domains and intelligence as covariates.

Potential confounds on neurocognition within the UHR group were investigated using Pearson or Spearman correlation analyses as appropriate (associations with global scores on psychopathological measures regarding general psychiatric symptoms, mania, depression, and negative symptoms). Welch's *t* test or Mann–Whitney test, as appropriate, compared domain and overall composite performances in subsamples of UHR individuals that (had) received medication vs. those that did not.

Two‐sided significance level was set to *α* ≤ 0.05. For ease of interpretation, the primary effect size reported for all between‐group comparisons of neurocognitive functioning is Cohen's *d* (Cohen, 1988). In ANCOVAs, we also report percentage reduction in *d* values after adjustment for each neurocognitive covariate. IBM SPSS Version 22.00 (Armonk, NY: IBM Corp) was used for all analyses.

## 3 RESULTS

### 3.1 Between‐group comparisons of demographic and clinical characteristics

Information on demographic and clinical variables in the UHR and healthy control group is summarized in Table 3. Groups were highly similar on basic demographic parameters, except controls having significantly more years of education. The UHR group had markedly elevated scores on all psychopathological measures.

**TABLE 3:** Demographic and clinical characteristics of the study participants

Measure^a^	Ultra‐high risk group (*n* = 50)	Healthy control group (*n* = 50)	*p*‐value^b^
Demographics			
Age, *M* (±*SD*), *Mdn* (range)	23.6 (4.6), 22 (19)	23.5 (4.4), 22 (20)	.99
Female gender, *n* (%)	28 (56.0)	28 (56.0)	1.00
Years of education, *M* (±*SD*)	13.0 (2.8)	14.1 (2.0)	.020*
Parental socioeconomic status^c^, A/B/C (%/%/%)	30/20/0 (60.0/40.0/0.0)	30/20/0 (60.0/40.0/0.0)	1.00
Race/ethnicity			1.00
1. White, *n* (%)	47 (94.0%)	46 (92.0%)	1.00
2. Asian, *n* (%)	3 (6.0%)	3 (6.0%)	
3. Mixed White/Asian, *n* (%)	0 (0.0%)	1 (2.0%)	
Psychopathology			
Ultra‐high risk intake groups			
1. Trait‐plus‐State Risk Factors, *n* (%)	29 (58.0)	—	—
2. Attenuated Psychotic Symptoms, *n* (%)	47 (94.0)	—	—
3. Brief Limited Intermittent Psychotic Symptoms, *n* (%)	3 (6.0)	—	—
First‐degree family history of psychotic disorder, *n* (%)	11 (22.0)	—	—
Diagnosis of schizotypal personality disorder, *n* (%)	22 (44.0)	—	—
Global psychiatric symptoms (BPRS‐E)^d^, *M* (±*SD*), *Mdn* (range)	46.2 (10.6), 44 (43)	26.0 (3.0), 25 (13)	≤.001^***^
Negative symptoms (SANS)^e^, *M* (±*SD*), *Mdn* (range)	1.8 (0.7), 2.0 (3.3)	0.1 (0.2), 0.0 (1.0)	≤.001^***^
Depressive symptoms (MADRS)^f^, *M* (±*SD*), *Mdn* (range)	18.4 (7.7), 17 (28.0)	1.0 (1.9), 0.0 (8.0)	≤.001^***^
Mania symptoms (YMRS)^g^, *M* (±*SD*), *Mdn* (range)	2.9 (3.0), 2 (10.5)	0.8 (1.2), 0.0 (5.0)	≤.001^***^
Functional level			
Psychosocial functioning (SOFAS)^h^, *M* (±*SD*), *Mdn* (range)	44.2 (7.2), 42 (38)	82.6 (3.5), 83 (20)	≤.001^***^
Medication			
Lifetime antipsychotic use, *n* (%)	12 (24.0)	—	—
Lifetime dose of haloperidol or equivalent (≤50 mg), *M* (±*SD*)	17.56 (13.04)	—	—
Current use of antidepressants, *n* (%)	16 (32.0)	—	—

^a^ *SD*, standard deviation; *M*, Mean; *Mdn*, median; *n*, subsample size.

^b^ Student's *t* test, Welch's *t* test, Mann–Whitney test, chi‐square test of independence, or Fisher's exact test was used, as appropriate.

^c^ Based on parents' income and level of education, parental socioeconomic status was categorized into three groups, A being the highest and C the lowest.

^d^ Brief Psychiatric Rating Scale Expanded Version (4.0); (∑ item 1–24).

^e^ Scale for the Assessment of Negative Symptoms; (∑global rating of Affective Flattening; global rating of Alogia; global rating of Avolition‐Apathy; global rating of Anhedonia‐Asociality)/4.

^f^ Montgomery‐Åsberg Depression Rating Scale; (∑ item 1–10).

^g^ Young Mania Rating Scale; (∑ item 1–11).

^h^ Social and Occupational Functioning Assessment Scale.

* *p* ≤ .05, **p ≤ .01, ***p ≤ .001: Significance levels.

### 3.2 Unadjusted between‐group differences in neurocognitive functioning

**FIGURE 1:**
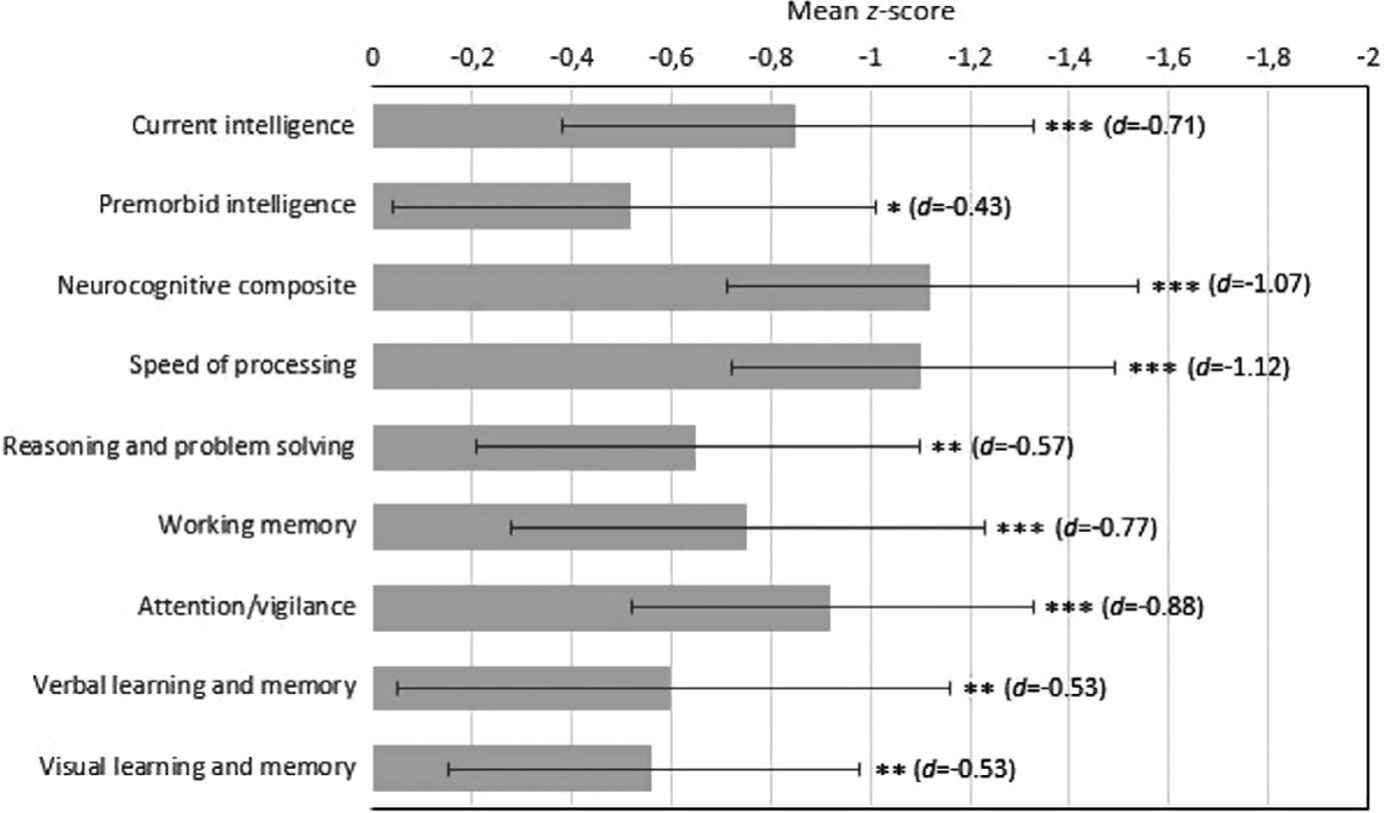
Neurocognitive functioning of the ultra‐high risk group (*n* = 50) presented as *z*‐score deficits relative to the healthy control group (*n* = 50) (with a mean of 0). Error bars indicate 95% confidence interval of the difference between group means. Significance levels: *p ≤ .05, **p ≤ .01, ***p ≤ .001. Cohen's *d* is provided in brackets

Mean or median neurocognitive performance for each measure across groups is summarized in Table S1. Results of primary unadjusted analyses on neurocognitive domain functioning in the two groups are presented in Figure 1. Markedly impaired neurocognitive functioning in the UHR group was indicated by a highly significant multivariate group effect in the MANOVA comparing all MATRICS domains combined, *F*(6,93) = 5.989, *p* ≤ .001; Wilks' Λ = 0.721, *d* = −1.24. Univariate group comparisons confirmed significantly lower performances across all domains (*d*s = −0.53 to −1.12). A two‐way mixed ANOVA with a Greenhouse–Geisser correction did not detect a significant group by domain interaction, *F*(4.414, 432.528) = 1.534, *p* = .19, *d* = −0.25. This suggests that there is no deviation from flatness in the UHR group's neurocognitive profile relative to healthy controls. It indicates that no domain score is significantly different from any of the other domain scores; thus, there is a nonselective pattern of neurocognitive impairment across the domains in the UHR group. The UHR individuals performed significantly worse on tests measuring premorbid and current intelligence.

### 3.3 Adjusted between‐group differences in neurocognitive domain functioning

The primary MANCOVA model controlling for speed of processing while comparing the five remaining domains showed that processing speed made the otherwise highly significant multivariate group difference nonsignificant, *F*(5,93) = 0.969, *p* = .44; Wilks' Λ = 0.950, *d* = −0.46, suggesting that this domain may account for significant group differences in all other domains. In post hoc MANCOVAs covarying each of the other domains, that is, attention/vigilance, *F*(5,93) = 2.896 *p* = .018; Wilks' Λ = 0.865, *d* = −0.79, working memory, *F*(5,93) = 3.787, *p* = .004; Wilks' Λ = 0.831, *d* = −0.90, reasoning and problem solving, *F*(5,93) = 5.249, *p* ≤ .001; Wilks' Λ = 0.780, *d* = −1.06, verbal learning and memory, *F*(5,93) = 5.444, *p* ≤ .001; Wilks' Λ = 0.774, *d* = −1.08, and visual learning and memory, *F*(5,93) = 5.482, *p* ≤ .001; Wilks' Λ = 0.772, *d* = −1.09, the multivariate group effect was not removed, suggesting that these domain deficits do not underlie group differences in other domains in general. A MANCOVA comparing all six domains between groups while controlling for estimated current intelligence was also significant, *F*(6,92) = 3.406, *p* ≤ .001; Wilks' Λ = 0.818, *d* = −0.94, suggesting that generalized domain impairment is not simply attributable to lower intelligence.

ANCOVAs comparing individual neurocognitive domains between groups while controlling separately for each other domain as well as intelligence were performed to explore multivariate test results in detail, as presented in Table 4. The primary ANCOVAs controlling processing speed eliminated significant group differences across all other domains, on average reducing unadjusted *d* values with two‐thirds (68%). Post hoc ANCOVAs controlling for each of the other domains and intelligence did not suggest the same global impact on domain functioning, with *d* values averagely being reduced from 20% to 48%.

**TABLE 4:** Magnitude of mean between‐group difference (Cohen's *d*) in neurocognitive domain functioning before and after adjustment for each domain and intelligence

Neurocognitive domain	Unadjusted domain group difference	Domain between‐group difference and percentage reduction after using each neurocognitive domain and intelligence as the covariate						
		Speed of processing	Attention/vigilance	Working memory	Reasoning and problem solving	Verbal learning and memory	Visual learning and memory	Current intelligence
Speed of processing	−1.12^***^		−0.75^***^ (33%)	−0.84^***^ (25%)	−0.98^***^ (13%)	−0.96^***^ (14%)	−0.97^***^ (13%)	−0.86^***^ (23%)
Attention/Vigilance	−0.88^***^	−0.39 (55%)		−0.59^**^ (33%)	−0.71^***^ (19%)	−0.77^***^ (13%)	−0.72^***^ (18%)	−0.67^**^ (24%)
Working memory	−0.77^***^	−0.33 (57%)	−0.42* (45%)		−0.53* (31%)	−0.62^**^ (19%)	−0.60^**^ (22%)	−0.48^* a^ (38%)
Reasoning and problem solving	−0.57^**^	−0.26 (54%)	−0.25 (56%)	−0.12 (79%)		−0.41* (28%)	−0.42* (26%)	−0.01 (98%)
Verbal learning and memory	−0.53^**^	−0.03 (94%)	−0.33 (38%)	−0.28 (47%)	−0.37 (30%)		−0.40 (25%)	−0.22 (58%)
Visual learning and memory	−0.53^**^	−0.11 (79%)	−0.18 (66%)	−0.23 (57%)	−0.37 (30%)	−0.39 (26%)		−0.31 (42%)
Mean percentage reduction in Cohen's *d*		68%	48%	48%	25%	20%	21%	47%

Note

One‐way analyses of covariance (ANCOVAs) comparing ultra‐high risk individuals (*n* = 50) and healthy controls (*n* = 50). Columns indicate neurocognitive variable used as the covariate, rows indicate neurocognitive domain used as the dependent variable. Blank spaces represent analyses where a domain would have been both dependent variable and covariate. Negative Cohen's *d* value signifies worse performance of the ultra‐high‐risk group. Reduction in *d* value after adjustment is shown within brackets as rounded percentage.

^a^ A significant interaction term (*p* = .015) between group and current intelligence indicated that the assumption of homogeneity of regression slopes was violated, and this result should therefore be interpreted cautiously.

* *p* ≤ .05, **p ≤ .01, ***p ≤ .001: Significance levels.

Given the hypothesized key role of speed of processing, other domains used as covariates might have eliminated significant group differences due to their shared variance with processing speed. We therefore carried out supplementary analyses, regressing each domain on speed of processing and using the standardized residuals as individual processing speed‐independent covariates in ANCOVAs comparing groups across domains (Ojeda et al., 2012). As shown in Table S2, none of these residual domains eliminated any significant domain group differences. Percentage reduction of unadjusted *d* values was now minor or even negligible. Supplementary, it was also assessed if the processing speed measures with a substantial motor component, when used as a composite covariate, were able to remove the significant between‐group differences across the other five neurocognitive domains. The revised processing speed composite encompassed three measures, that is, the BACS Token Motor Task, BACS Symbol Coding, and Trail Making Test A, thus excluding the two nonmotor BACS Verbal Fluency tasks originally included in the broader processing speed domain. When this revised processing speed composite was included in a MANCOVA as a covariate while comparing the five other neurocognitive domains between the two groups, the significant multivariate between‐group difference was eliminated, *F*(5,93) = 1.271, *p* = .28; Wilks' Λ = 0.936, suggesting that the revised speed domain may account for significant group differences across the other domains. Post hoc univariate ANCOVAs comparing each neurocognitive domain between the two groups while controlling for the revised processing speed composite were next performed. The results suggested that covarying the revised processing speed domain eliminates the significant between‐group difference across all individual neurocognitive domains except attention/vigilance. For detailed information on these univariate analyses, see Table S3.

### 3.4 Confounder analyses

No significant associations between psychopathological measures and neurocognitive domain or overall neurocognitive composite functioning were observed within the UHR group, as shown in Table S4. There were also no significant differences in neurocognitive functioning between subsamples of UHR individuals that (had) received medication vs. those that did not.

## 4 DISCUSSION

We examined neurocognitive domain functioning in a sample of UHR individuals compared with demographically well‐matched healthy controls, focusing on two cardinal features characterizing neurocognition in schizophrenia, that is, a generalized deficit profile with slowed neurocognitive speed at its core. Our study essentially confirmed our two hypotheses; the UHR group was globally impaired across all neurocognitive domains, and reduced speed of processing appeared to account for all significant domain group differences.

The finding that the UHR group was globally neurocognitively impaired is in agreement with meta‐analyses (e.g., Giuliano et al., 2012; Hauser et al., 2017) and individual studies (e.g., Lencz et al., 2006; Ohmuro et al., 2015) demonstrating widespread impairment across, more or less, all measured domains in UHR individuals. It also corresponds to findings in psychotic disorders in general (Reilly & Sweeney, 2014) and schizophrenia (Schaefer et al., 2013). Speed of processing yielded the numerically largest effect size, in accordance with the most recent and most comprehensive (Hauser et al., 2017) meta‐analyses on neurocognition in the UHR state (Hauser et al., 2017; Zheng et al., 2018) as well as schizophrenia (Schaefer et al., 2013), but it was not significantly larger than any of the other neurocognitive domain effect sizes. The UHR group demonstrated a flat deficit profile, and medium to large effect sizes across all domains contribute to the notion of a broad‐based neurocognitive deficit (Mesholam‐Gately et al., 2009).

Our UHR sample, however, appears to be more neurocognitively impaired than many other UHR samples. Generalized impairment does not characterize all UHR samples (Pukrop & Klosterkötter, 2010); some studies have reported no significant deficits (e.g., Thompson et al., 2012) or only deficits in one or some measured domains (e.g., Niendam et al., 2006; Woodberry et al., 2010). Impairment in estimated current intelligence (*d* = −0.71) was also more pronounced in our UHR sample than in meta‐analyses, with effect sizes ranging from *g* = −0.21 (Hauser et al., 2017) to *d* = −0.53 (Giuliano et al., 2012). Furthermore, our sample demonstrated neurocognitive deficits of larger magnitudes than hypothesized, with a substantial composite effect size (*d* = −1.07). Even though many UHR studies have detected comparable or even larger effect sizes across domains (e.g., Frommann et al., 2011; Ohmuro et al., 2015; Simon et al., 2007) and a UHR meta‐analysis (Zheng et al., 2018) based only on studies using the MATRICS Consensus Cognitive Battery has reported effect sizes similar to the ones found in our study, meta‐analyses have typically reported effects sizes in the small to medium range (e.g., Bora et al., 2014; Giuliano et al., 2012; Hauser et al., 2017). We suspect that our findings may reflect that only UHR individuals with sustained low or significant drop in functioning were included in the study, as per inclusion criterion. In most other UHR studies, the functional impairment criterion is only part of the Vulnerability (Trait and State Risk) Group, but not of the Attenuated Psychotic Symptoms (APS) Group nor of the Brief Limited Intermittent Psychotic Symptoms (BLIPS) Group. A link between neurocognitive deficits and functional impairment has been documented in a UHR meta‐analysis (Bora et al., 2014) as well as systematic review (Cotter et al., 2014) and is well‐established in schizophrenia (Fett et al., 2011; Green et al., 2000). The difference in mean SOFAS scores between the UHR and healthy control group corresponded to *g* = −6.78, which is substantially larger than the meta‐analytic mean effect size of *g* = −3.01 characterizing UHR individuals' low functioning (Fusar‐Poli et al., 2015). Functional impairment in our UHR sample is more comparable to that of first‐episode psychosis individuals in most studies comparing these individuals to UHR individuals (e.g., Eastvold et al., 2007).

Both primary multivariate and univariate covariate models suggested that overarching neurocognitive slowing may account for significant between‐group differences across all domains and may therefore represent a critical factor in neurocognitive processing inefficiency in UHR individuals. Even though processing speed is not the exclusive source of all observed between‐group variability across neurocognitive domains, a substantial portion of this variance appears to be shared with this domain. To strengthen the assertion of a unique effect of decreased neurocognitive speed on generalized domain impairment, post hoc covariate analyses suggested that neither of the other neurocognitive domains nor estimated current intelligence used as covariates had the same global impact on domain functioning, and covarying each of the other domains after removing their shared variance with processing speed revealed a pattern of group effects identical to that of the original unadjusted analyses. The UHR group's inferior neurocognitive performances across domains may therefore be secondary to primary neurocognitive slowing and reflect a relatively parsimonious neurocognitive architecture. The overall pattern of results is noteworthy considering that the UHR group demonstrated a flat deficit profile, indicating that processing speed is not disproportionately impaired.

It is well‐documented that performances on a broad variety of neurocognitive tasks and composite domains share considerable common variance, both in healthy populations (Carroll, 1993) and schizophrenia (Dickinson & Gold, 2008a). Multiple studies have considered the influence of intelligence on neurocognitive impairment in UHR individuals and found that it may account for or eliminate some, but not all, significantly impaired performances across specific neurocognitive tasks or domains (e.g., Seidman et al., 2016), in accordance with our results. However, to our knowledge, only few UHR studies (Frommann et al., 2011; Koutsouleris et al., 2010; Woodberry et al., 2010) have considered the potential influence of specific neurocognitive domain or task performances on between‐group neurocognitive functioning, and it remained to be determined whether a distinct processing speed domain may significantly contribute to deficits across a broad spectrum of neurocognitive domains. Our findings are in general agreement with a growing body of schizophrenia studies showing that decreased processing speed, to a significant degree, may underlie impairment in an array of neurocognitive domains (e.g., Andersen et al., 2013; Brébion et al., 2014; Fuller et al., 2005; Hartman et al., 2003; Holthausen et al., 2003; Kochunov et al., 2017; Ojeda et al., 2012; Rodríguez‐Sánchez et al., 2007; Salamé et al., 1998; Sanfilipo et al., 2002; Schatz, 1998). Our findings likewise coincide with similar research on aging and other neurocognitively impaired populations (e.g., Butters & et al., 2004; Lee et al., 2012; Liebel et al., 2017; McGrath et al., 2011; Salthouse, 1996; Su et al., 2015).

The influence of speed of processing on other domains may be obvious in the case of attention/vigilance that requires speeded response to a considerable degree. Still, most neurocognitive variables included in the other domains are untimed, indicating that the processing speed influence on these domains is not just secondary to a common procedural factor. Processing speed, as a nontask‐specific mental capacity, may impose limits on a broad diversity of neurocognitive processing operations and therefore constitute a rate‐limiting factor for performances (Kail & Salthouse, 1994). Slower speed of executing a variety of neurocognitive processing operations results in less completion of processing in a given amount of time and reduces the amount of simultaneously available information when needed (Salthouse, 1996). Therefore, although this remains speculations, UHR individuals may not be able to complete and coordinate all the information processing needed for adequate performances within a given amount of time.

Supplementary analyses showed that a revised processing speed composite only including the motor‐based tasks, i.e. excluding verbal fluency, when used as the covariate, eliminated significant between‐group differences across all neurocognitive domains except for the attention/vigilance domain, thus replicating the original findings for four out of five domains. This may raise the question as to whether the motor‐based and revised processing speed composite, as compared to the original broader processing speed domain construct, is less efficient at explaining the significant between‐group difference in the attention/vigilance domain. It should however be noted that the supplementary analyses confirmed the original findings in the remaining neurocognitive domains. Also, according to the MATRICS recommendations, verbal fluency is included in the processing speed domain as factor analyses have revealed this neurocognitive function to most commonly load on the processing speed factor (Nuechterlein et al., 2004). Overall, the results suggest that a heterogenous processing speed composite, including both speeded motor and language tasks, may explain significant between‐group differences in only one additional domain, that is, attention/vigilance, as compared to the motor‐based and revised processing speed composite. This may suggest that there is less of an overlap between the revised processing speed composite and the attention/vigilance domain, perhaps because the excluded verbal fluency tasks tap more into executive attention resources.

The MATRICS speed of processing domain encompasses both basic motor and perceptual components as well as executive control, that is, verbal fluency (Nuechterlein et al., 2004). Coding and fluency tasks require rapid and smooth coordination of a complex assembly of basic neurocognitive operations, including visual scanning, motor abilities, flexibility, and neurocognitive control. Compromised ability to perform such tasks adequately may therefore reflect insufficient coordination or inability to efficiently and rapidly connect spatially distributed and interconnected brain regions, that is, deficits in connectivity (Dickinson et al., 2007). Our study may suggest a systemic perspective on neurocognitive impairment in the UHR state (Dickinson & Harvey, 2009; Kelleher et al., 2013), including reduced white matter integrity (Krakauer et al., 2017; Kristensen et al., 2019). In this regard, it should be noticed that multiple studies have demonstrated decreased processing speed to be associated with reduced white matter integrity in schizophrenia (Karbasforoushan et al., 2015; Kochunov et al., 2017; Peng et al., 2020), first‐episode psychosis (Faria et al., 2019), recent onset psychosis (Szeszko et al., 2018), aging (Kerchner et al., 2012), and other neurocognitively impaired populations (e.g., Segura et al., 2010; Soria‐Pastor et al., 2008; Yu et al., 2012).

Our findings have other important research and clinical implications. Processing speed appears to be particularly important to measure in UHR individuals, also for screening purposes (González‐Blanch et al., 2011), because it may capture the generalized impairment. From a treatment perspective, our study may provide a rationale for targeting this apparently domain‐general neurocognitive mechanism, using behavioral and/or pharmacological interventions to boost neurocognitive processing efficiency (Brébion et al., 2014; Cassetta & Goghari, 2016; Cassetta et al., 2019; Takeuchi & Kawashima, 2012). Thus, a double‐blind randomized clinical trial has shown that UHR individuals receiving processing speed training exhibit improvement not only in processing speed but also in social functioning (Choi et al., 2017).

## 5 LIMITATIONS

Our findings should be interpreted cautiously in the context of several well‐acknowledged limitations. First, construct validity of the speed of processing domain may be questioned (Carter & Barch, 2007). Given its multi‐componential nature, it is likely simultaneously sensitive to and taps multiple neurocognitive functions (Dickinson et al., 2007). It may therefore be difficult to classify and isolate a distinct, unitary speed domain, and future studies need to examine the neurocognitive underpinnings of speed of processing in UHR individuals in more detail (Chiaravalloti et al., 2003; Knowles et al., 2012). Nevertheless, the domain‐general and broad‐ranging nature of processing speed is likely quintessential to understanding its key role in the neurocognitive architecture of UHR individuals. Our categorization of this domain also followed the MATRICS recommendations (Nuechterlein et al., 2004), and five outcome variables were included in the speed composite to enhance both validity and reliability. Concerning the multi‐componential nature of processing speed, it should also be noted that neurocognitive tests of processing speed typically lack the precision of determining the more specific neurocognitive component operations involved (Dickinson et al., 2007). Furthermore, there is no agreed upon consensus as to what components may constitute processing speed (Low et al., 2017), and an analysis of the potential components of this domain was not performed in the present study. Thus, for instance, response selection was not specifically identified as part of the processing speed, even though this may be an important component to take into consideration (Woodward et al., 2013, 2014). Future UHR studies should include measures that make it possible to fractionate processing speed into its distinct components, just as using cognitive neuroscience‐based approaches would allow for examining relevant components, including the response selection stage of information processing (Woodward et al., 2013, 2014). Second, the two neurocognitive domains demonstrating the numerically smallest effect sizes, verbal as well as visual learning and memory, each consisted of only one outcome variable, and this may have caused these domains to be less sensitive to group differences. It has been suggested that at least three outcome variables should be included in a domain composite to ensure adequate psychometric quality (Kenny et al., 1998), and UHR meta‐analytic results indicate that the verbal learning and memory domain is impaired at the same level as processing speed (Hauser et al., 2017). To add to this limitation, neurocognitive tests were generally not psychometrically matched in our study, and it is uncertain if discriminating power was comparable across tests (Chapman & Chapman, 1973). Outcome variables associated with the processing speed domain may have been somewhat better at capturing an underlying generalized performance deficit than were outcome variables associated with other domains (Dickinson & Harvey, 2009). Still, we used well‐validated tests and outcome variables classified according to the MATRICS recommendations. Effect sizes across neurocognitive domains were also comparable in the UHR group given that an essentially flat deficit profile was detected, at least indicating that out study did not artifactually produce differential domain deficits. Third, slowed neurocognitive speed may be a robust correlate of psychopathology in general (Nigg et al., 2017) and thus a nonspecific marker of mental illness (Pukrop et al., 2007); a digit symbol substitution test has failed to detect significant differences between UHR individuals and psychiatric controls (Ilonen et al., 2010; Lindgren et al., 2010). Thus, it is a limitation that the comorbidity in the UHR group was not addressed in the present study, which makes it difficult to address the specificity of the observed impairment in processing speed and of its effects on the between‐group differences in other neurocognitive domains. Fourth, we only used cross‐sectional data, and even if tests covering speed of processing may be relevant for psychosis prediction (Hauser et al., 2017; Studerus et al., 2016; Velthorst et al., 2019), the potential key role of slowed neurocognitive speed in UHR individuals with later transition to psychosis needs to be examined in depth in future longitudinal studies. Fifth, we used covariate analyses that have been extensively used in studies similar to ours, including UHR studies controlling neurocognitive functioning for specific domains (e.g., Frommann et al., 2011) and/or intelligence (e.g., Seidman et al., 2016). Such analyses may be reasonable for descriptive model building (Tabachnick & Fidell, 2007), and only in this way was it possible to examine the extent to which impairment in one domain might reflect impairment in another domain (Esbjørn et al., 2006). Still, hypothetical group matching does not allow for inferring causality and remains debatable (e.g., Dennis et al., 2011). Future research should (ideally) be designed to examine if a causal relationship between decreased processing speed and impairment in other domains can be established. Sixth, only around one‐third of UHR individuals has been found to develop psychosis during follow‐up (Schultze‐Lutter et al., 2015) and even though the vast majority develop schizophrenia spectrum disorders, not all do (Fusar‐Poli et al., 2013). Apparent neurocognitive commonalities between the UHR state and schizophrenia should therefore be treated cautiously. Seventh, comparisons of neurocognitive functioning in UHR individuals relative to healthy controls were not controlled for the potential difference in alcohol and drug use behaviors. Such behaviors have negative effects on neurocognition and may therefore constitute a confounding factor (Broyd et al., 2016; Potvin et al., 2018; Scott et al., 2018; Stavro et al., 2013). Unfortunately, such potential effects have often not been considered in UHR studies. It may be informative for subsequent UHR studies to examine and account for the potential effects of alcohol and drug use behaviors on neurocognition, including processing speed.

## 6 CONCLUSION

Our study found evidence that decreased speed of processing may account for the global impairment across other neurocognitive domains in UHR individuals. Future studies are needed to examine if findings can be replicated in UHR samples with varying characteristics. We hope the study will stimulate further UHR research designed to understand the possible contribution of general neurocognitive slowing to broadly impaired neurocognitive functioning. Future studies should also further assess the associations between processing speed and social functioning (Carrión et al., 2011) as well as social cognitive domain functioning (Glenthøj et al., 2018) in the UHR population.

## CONFLICT OF INTEREST

Dr. Glenthøj is the leader of a Lundbeck Foundation Center of Excellence for Clinical Intervention and Neuropsychiatric Schizophrenia Research (CINS), which is partially financed by an independent grant from the Lundbeck Foundation based on international review and partially financed by the Mental Health Services in the Capital Region of Denmark, the University of Copenhagen, and other foundations. Her group has also received a research grant from Lundbeck A/S for another independent investigator‐initiated study. All grants are the property of the Mental Health Services in the Capital Region of Denmark and administrated by them. She has no other conflicts to disclose. All other authors declare that there are no conflicts of interest in relation to the subject of this study.

## AUTHOR CONTRIBUTIONS

Lasse Randers: Conceptualization, Data curation, Formal analysis, Funding acquisition, Investigation, Methodology, Project administration, Writing of the original draft, Review and editing of the manuscript. Jens Richardt M Jepsen: Conceptualization, Data curation, Formal analysis, Methodology, Writing of the original draft, Review and editing of the manuscript. Birgitte Fagerlund: Conceptualization, Data curation, Methodology, Resources, Writing of the original draft, Review and editing of the manuscript. Dorte Nordholm: Conceptualization, Data curation, Funding acquisition, Investigation, Methodology, Project administration, Review and editing of the manuscript. Kristine Krakauer: Data curation, Investigation, Project administration, Review and editing of the manuscript. Carsten Hjorthøj: Formal analysis, Review and editing of the manuscript. Birte Glenthøj: Funding acquisition, Resources, Review and editing of the manuscript. Merete Nordentoft: Conceptualization, Funding acquisition, Methodology, Resources, Review and editing of the manuscript.

### Peer Review

The peer review history for this article is available at https://publons.com/publon/10.1002/brb3.1962.

## Supporting information

Table S1

Table S2

Table S3

Table S4

## Data Availability

The data that support the findings of this study are available on request from the corresponding author. The data are not publicly available due to privacy or ethical restrictions.
