# Supplementary material for: Generalized neurocognitive impairment in individuals at ultra‐high risk for psychosis: The possible key role of slowed processing speed
Source: Brain Behav. 2021 Jan 23;11(3):e01962. doi: 10.1002/brb3.1962 (PMC7994693; doi:10.1002/brb3.1962)
Supplement: Supplementary file 1 — Table S1 [file BRB3-11-e01962-s002.docx]

| Test | Outcome variable | Raw score mean ^₸^  (standard deviation) | | Raw score median ^ǂ^  (range) | |
| --- | --- | --- | --- | --- | --- |
|  |  | UHR group  (*n* = 50) | HC  group  (*n* = 50) | UHR  group  (*n* = 50) | HC  group  (*n* = 50) |
| Current intelligence (estimated) | | | | | |
| WAIS-III Vocabulary | Total score ^§^ |  |  | 30.00  (17.00 – 58.00) | 37.50  (21.00 – 58.00) |
| WAIS-III Similarities | Total score ^§^ |  |  | 24.00  (14.00 – 31.00) | 26.00  (14.00 – 32.00) |
| WAIS-III Block Design | Total score ^§^ |  |  | 52.00  (14.00 – 68.00) | 57.00  (30.00 – 68.00) |
| WAIS-III Matrix Reasoning | Total score ^§^ |  |  | 21.50  (10.00 – 26.00) | 22.00  (14.00 – 26.00) |
| Speed of processing | | | | | |
| BACS Verbal Fluency  Category Instances | Total number of correct words generated | 27.30  (6.90) | 31.32  (6.26) |  |  |
| BACS Verbal Fluency  Controlled Oral Word Association Test | Total number of correct words generated | 28.86  (6.88) | 34.58  (7.69) |  |  |
| BACS Token Motor Task | Total number of tokens correctly placed | 74.52  (10.14) | 80.20  (8.97) |  |  |
| BACS Symbol Coding | Total number of written symbols correctly matched with digits | 57.88  (10.69) | 66.50  (11.52) |  |  |
| Trail Making Test A | Time to completion ^ǂǂ^ |  |  | 23.00  (15.00 – 40.00) | 18.00  (12.00 – 46.00) |
| Working memory | | | | | |
| BACS Digit Sequencing Task | Total number of correctly ordered sequences of digits | 19.44  (4.54) | 21.94  (3.53) |  |  |
| CANTAB Spatial Working Memory | Total number of between-search errors ^¶^ |  |  | 9.00  (0.00 – 76.00) | 4.00  (0.00 – 60.00) |
| Trail Making Test B | Time to completion ^¶^ |  |  | 61.00  (34.00 – 112.00) | 46.00  (22.00 – 78.00) |
| CANTAB Spatial Span | Span length ^§^ |  |  | 7.00  (3.00 – 9.00) | 8.00  (3.00 – 9.00) |
| Verbal learning and memory | | | | | |
| BACS List learning | Total recall across five trials | 49.46  (9.42) | 54.00  (7.47) |  |  |
| Visual learning and memory | | | | | |
| CANTAB Delayed Matching to Sample | Total number of correct responses (all delays) ^¶^ |  |  | 26.00  (17.00 – 30.00) | 28.00  (21.00 – 30.00) |
| Reasoning and problem solving | | | | | |
| BACS Tower of London | Total number correct | 18.60  (3.07) | 19.44  (1.79) |  |  |
| CANTAB Stockings of Cambridge | Problems solved in minimum moves ^§^ |  |  | 10.00  (5.00 – 12.00) | 10.00  (6.00 – 12.00) |
| CANTAB Spatial Working Memory | Strategy score ^¶^ |  |  | 28.00  (18.00 – 40.00) | 22.00  (18.00 – 35.00) |
| CANTAB Intra/Extra Dimensional Set Shift | Total errors adjusted ^ǂǂ^ |  |  | 10.00  (5.00 – 62.00) | 9.00  (6.00 – 62.00) |
| CANTAB Intra/Extra Dimensional Set Shift | Total extra-dimensional stage errors ^ǂǂ^ |  |  | 3.00  (0.00 – 32.00) | 2.00  (0.00 – 32.00) |
| Trail Making Test B - Trail Making Test A | Difference between time to completion of Trail  Making Test B and A ^§^ |  |  | 38.00  (13.00 – 85.00) | 26.50  (-1.00 – 63.00) |
| Attention/vigilance | | | | | |
| CANTAB Rapid Visual Information Processing | Signal detection measure A' ^₸₸, §§^ |  |  | 0.95  (0.82 – 1.00) | 0.97  (0.89 – 1.00) |
| CANTAB Reaction Time  Simple Reaction Time | Response latency (simple) ^ǂǂ^ |  |  | 306.44  (241.44 – 446.22) | 288.11  (238.50 – 397.56) |
| CANTAB Reaction Time  5-Choice Reaction Time | Response latency (5-choice) ^ǂǂ^ |  |  | 342.00  (271.63 – 496.75) | 319.63  (253.75 – 444.14) |

UHR = ultra-high risk; HC = healthy control; WAIS-III = Wechsler Adult Intelligence Scale–Third Edition; BACS = Brief Assessment of Cognition in Schizophrenia; CANTAB = Cambridge Neuropsychological Test Automated Battery.

^₸^ The mean neurocognitive group performances are provided when the neurocognitive variables are normally distributed and therefore

have not been transformed.

^ǂ^ The median neurocognitive group performances are provided when the neurocognitive variables in question have been transformed

due to non-normality or skewness.

^§^ Square root transformation.

^¶^ Lg10 transformation.

^₸₸^ LnGamma transformation.

^ǂǂ^ Reciprocal transformation.

^§§^ One outlier data point was removed and replaced with the group mean in the healthy control group.
