# Supplementary material for: Generalized neurocognitive impairment in individuals at ultra‐high risk for psychosis: The possible key role of slowed processing speed
Source: Brain Behav. 2021 Jan 23;11(3):e01962. doi: 10.1002/brb3.1962 (PMC7994693; doi:10.1002/brb3.1962)
Supplement: Supplementary file 2 — Table S2 [file BRB3-11-e01962-s004.docx]

**TABLE S2** Magnitude of mean between-group difference (Cohen’s d) in neurocognitive domain functioning before and after adjustment for each domain regressed on speed of processing

| Neurocognitive domain | Unadjusted domain group difference | Domain between-group difference and percentage reduction after adjustment for each neurocognitive domain regressed on speed of processing | | | | |
| --- | --- | --- | --- | --- | --- | --- |
|  |  | Residual of  attention/ vigilance | Residual of  working memory | Residual of  reasoning and problem solving | Residual of  verbal learning and memory | Residual of  visual learning and memory |
| Attention/  vigilance | -0.88*** |  | -0.84***  (5%) | -0.86***  (2%) | -0.88***  (0%) | -0.88***  (0%) |
| Working  memory | -0.77*** | -0.71***  (8%) |  | -0.76***  (1%) | -0.77***  (0%) | -0.77***  (0%) |
| Reasoning and problem solving | -0.57** | -0.50*  (12%) | -0.49*  (14%) |  | -0.57**  (0%) | -0.56**  (2%) |
| Verbal learning and memory | -0.53** | -0.53**  (0%) | -0.50*  (6%) | -0.50*  (6%) |  | -0.53*  (0%) |
| Visual learning and memory | -0.53** | -0.46*  (13%) | -0.47*  (11%) | -0.50*  (6%) | -0.53*  (0%) |  |
| Mean percentage reduction in Cohen’s *d* |  | 8% | 9% | 4% | 0% | 1% |

One-way analyses of variance (ANCOVAs) comparing ultra-high risk individuals (*n* = 50) and healthy controls (*n* = 50). Columns indicate neurocognitive domain used as the covariate, rows indicate domain used as the dependent variable. Blank spaces represent analyses where a domain would have been both dependent variable and covariate. Negative Cohen’s *d* value signifies worse performance of the ultra-high risk group. Reduction in *d* value after adjustment is shown within brackets as rounded percentage. Significance levels: **p*≤0.05, ***p*≤0.01, ****p*≤0.001.
