# Supplementary material for: Generalized neurocognitive impairment in individuals at ultra‐high risk for psychosis: The possible key role of slowed processing speed
Source: Brain Behav. 2021 Jan 23;11(3):e01962. doi: 10.1002/brb3.1962 (PMC7994693; doi:10.1002/brb3.1962)
Supplement: Supplementary file 3 — Table S3 [file BRB3-11-e01962-s003.docx]

**TABLE S3** Between-group univariate comparisons (ANCOVAs) of neurocognitive functioning in five domains in the ultra-high risk and healthy control group using the revised processing speed composite as the covariate

| Neurocognitive domain | *F*-value | *p*-value |
| --- | --- | --- |
| Attention/vigilance | *F* (1,97) = 5.014 | *p* = 0.027* |
| Working memory | *F* (1,97) = 3.031 | *p* = 0.09 |
| Reasoning and problem solving | *F* (1,97) = 1.647 | *p* = 0.20 |
| Verbal learning and memory | *F* (1,97) = 0.632 | *p* = 0.43 |
| Visual learning and memory | *F* (1,97) = 0.452 | *p* = 0.50 |

One-way analyses of variance (ANCOVAs) comparing ultra-high risk individuals (*n*=50) and healthy controls (*n*=50).

Significance level: **p*≤0.05.
