# Supplementary material for: Generalized neurocognitive impairment in individuals at ultra‐high risk for psychosis: The possible key role of slowed processing speed
Source: Brain Behav. 2021 Jan 23;11(3):e01962. doi: 10.1002/brb3.1962 (PMC7994693; doi:10.1002/brb3.1962)
Supplement: Supplementary file 4 — Table S4 [file BRB3-11-e01962-s001.docx]

**TABLE S4** Pearson/Spearman correlation^₸^ matrix for neurocognitive variables with psychopathology variables within the ultra-high risk group

| Neurocognitive variable | Global psychiatric symptoms (BPRS-E) ^ǂ^ | Mania symptoms (YMRS) ^§^ | Depressive symptoms (MADRS) ^¶^ | Negative symptoms (SANS) ^₸₸^ |
| --- | --- | --- | --- | --- |
| Current intelligence ^ǂǂ^ | *r_s_*(50) = 0.080  *p* = 0.58 | *r_s_*(50) = 0.146  *p* = 0.31 | *r*(50) = 0.090  *p* = 0.53 | *r*(50) = 0.080  *p* = 0.58 |
| Composite ^§§^ | *r_s_*(50) = -0.110  *p* = 0.448 | *r_s_*(50) = -0.025  *p* = 0.86 | *r*(50) = -0.045  *p* = 0.76 | *r*(50) = -0.144  *p* = 0.32 |
| Speed of  Processing | *r_s_*(50) = -0.054  *p* = 0.71 | *r_s_*(50) = 0.025  *p* = 0.87 | *r*(50) = 0.085  *p* = 0.56 | *r*(50) = -0.219  *p* = 0.13 |
| Attention/  Vigilance | *r_s_*(50) = -0.011  *p* = 0.94 | *r_s_*(50) = 0.021  *p* = 0.89 | *r*(50) = 0.031  *p* = 0.83 | *r*(50) = -0.193  *p* = 0.18 |
| Working  Memory | *r_s_*(50) = -0.244  *p* = 0.09 | *r_s_*(50) = -0.236  *p* = 0.10 | *r*(50) = -0.178  *p* = 0.22 | *r*(50) = 0.054  *p* = 0.71 |
| Reasoning and problem solving | *r_s_*(50) = -0.087  *p* =0.55 | *r_s_*(50) = 0.057  *p* = 0.69 | *r*(50) = -0.107  *p* = 0.46 | *r*(50) = -0.065  *p* = 0.66 |
| Verbal learning and memory | *r_s_*(50) = -0.003  *p* = 0.99 | *r_s_*(50) = 0.088  *p* = 0.54 | *r*(50) = 0.048  *p* = 0.74 | *r*(50) = -0.044  *p* = 0.76 |
| Visual learning and memory | *r_s_*(50) = -0.124  *p* = 0.39 | *r_s_*(50) = -0.129  *p*=0.37 | *r*(50) = -0.066  *p* = 0.65 | *r*(50) = -0.130  *p* = 0.37 |

^₸^ *r* refers to the Pearson correlation coefficient, whereas *r_s_* refers to the Spearman rank correlation coefficient.

^ǂ^  Brief Psychiatric Rating Scale Expanded Version (4.0); (∑ item 1-24). ^ǂ^

^§^ Young Mania Rating Scale; (∑ item 1-11).

^¶^ Montgomery-Åsberg Depression Rating Scale; (∑ item 1-10).

^₸₸^ Scale for the Assessment of Negative Symptoms; (∑global rating of Affective Flattening; global rating of Alogia; global rating of Avolition-Apathy; global rating of Anhedonia-Asociality) / 4.

^ǂǂ^  Estimation based on four subtests from the Wechsler Adult Intelligence Scale, Third Edition; Vocabulary, Similarities, Block Design and Matrix Reasoning.  ^§§^ Based on 20 neurocognitive outcome variables categorized according to the six neurocognitive Measurement and Treatment Research to Improve Cognition in Schizophrenia (MATRICS) domains.
